# The functional effect of 3D-printing individualized orthosis for patients with peripheral nerve injuries: Three case reports

**DOI:** 10.1097/MD.0000000000019791

**Published:** 2020-04-17

**Authors:** Dong-Sik Chae, Da-Ham Kim, Kyung-Yil Kang, Doo-Young Kim, Si-Woon Park, Sung-Jun Park, Jae-Hyung Kim

**Affiliations:** a Department of Orthopedic Surgery; b Department of Physical Medicine and Rehabilitation, Catholic Kwandong University International St Mary's Hospital, Incheon; c 3D Printing Center, Korea National University of Transportation, Chungju-si, Chungbuk, Republic of Korea.

**Keywords:** 3-dimensional printing, orthosis, peripheral nerve injury

## Abstract

**Rationale:**

In the medical field, the use of 3-dimensional (3D) printing is increasing explosively and it is especially widespread in the clinical application of fabricating orthosis. Advantages of 3D-printed orthosis compared to conventional ones include its lower cost, easier modification, and faster fabrication. The 3D-printing technique makes it possible for physicians to easily create individual-tailored products. Recently, many kinds of orthosis through 3D printing have been studied and used. The knee orthosis, ankle-foot orthosis, wrist orthosis, hand orthosis, and foot orthotics are examples used in the rehabilitation fields of orthotics. We reported 3 cases of 3D-printed orthoses in patients with peripheral nerve injuries.

**Patients concerns:**

In spite of the rapid development of the clinical use of 3D printing, to our knowledge, its application to patients with peripheral nerve injuries has not yet been reported. Two patients suffered from upper limb problems and 1 patient had a foot drop associated with peripheral nerve injury.

**Diagnosis:**

Three patients diagnosed with median neuropathy, ulnar neuropathy, and right lower lumbar radiculopathy, respectively, by electromyography.

**Interventions:**

Herein we present 3 case reports of patients with peripheral nerve injuries whose orthotic needs were fulfilled with the application of 3D-printed wrist orthosis and ankle-foot orthosis.

**Outcomes:**

For hand function evaluation, we assessed the Jebsen–Taylor hand function test. Grasp and pinch powers were assessed by a hand dynamometer before and after orthosis application. For lower limb functional evaluation, we used a 6-minute walking test and modified Emory Functional Ambulation Profile for ambulatory function.

**Lessons:**

The 3D-printed orthosis could help functional improvement in patients with peripheral nerve injuries.

## 1 Introduction

Peripheral nerve injuries result in possible motor loss and subsequent muscle imbalance, which could create functional loss secondary to adaptive contractile and no contractile tissue shortening or lengthening. To achieve functional gain or prevent functional decrease, patients with peripheral nerve injury can use various orthotic devices. The objectives of orthosis for the patients with peripheral nerve injury include protection of injured tissues, improvement of a healing environment, prevention or minimization of contracture formation, acting as a substitute for lost motor function, and facilitation and improvement of functional daily activities. The orthosis needs simplicity, cost accountability, flexibility, and sustainability in the attainment of orthosis effectiveness. These orthoses can be practical and can increase a patient's satisfaction and compliance.^[1]^ The ideal orthosis should be comfortable, lightweight, functional, easy to don and doff, and aesthetically pleasing to the patient while performing the function for which it was intended. There are many kinds of peripheral nerve injuries. Therefore, for compatible and functional orthosis, individualized orthosis should be required for the patients with peripheral nerve injuries.

Three-dimensional (3D) printing has been widely used in medical fields, and its use is growing explosively. The 3D printers can produce easily modifiable objects without any fixed moldings, which make the objects unique. Many kinds of orthotics through 3D printing have been studied and used. Knee orthosis, ankle-foot orthosis (AFO), wrist orthosis, hand orthosis, and foot orthotics are examples used in the rehabilitation field of orthotics.^[2–6]^ The 3D printing makes it possible for physicians to easily create patient-tailored products. To the best of our knowledge, this report is the 1st study of using 3D-printed orthosis in patients with peripheral nerve injuries. We investigate the functional effect of a patient-specific manufactured orthosis using 3D printing for patients with peripheral nerve injury.

## 2 Materials and methods

### 2.1 Manufacturing process for 3D-printing orthosis

We used thin-slice computed tomography (CT) images (1 mm) to form external contours of each body part for applied orthotics. After acquisition of the CT scan, a series would then be imported into MIMICS Medical v17 (Materialize, Leuven, Belgium), a 3D reconstruction software that produces 3D data from the CT scan. Then, the component is exported. We designed the 3D-printed orthosis using Geomagic Freeform software (3D SYSTEM Corp, Santa Clara, USA). The thickness of the orthosis was set to be 1.8 to 2.0 mm. The inner surface of the orthosis was separated 1 mm from the 3D surface images, which made the orthosis well ventilated and drained. Based on the prepared design, the orthosis was printed using a fused filament fabrication FINEBOT Z420 3D printer (TPC Mechatronics, Inc, Incheon, Korea). We used thermoplastic polyurethane filament as the output orthotic material, and the output surface directly touching the skin was smoothed through a postprinting process, which removed by-products used as supporting structures during the fused filament fabrication-type printing process. We performed this postprinting process manually using a grinding machine and pincers, which were used to remove the support materials from the printed orthosis, and we used grinding machine to smooth the surface of the orthosis. There was no additional manual adjustment of the orthosis after the postprinting process except to attach Velcro strap and ankle joint area re-enforcement.

### 2.2 Functional and satisfaction assessment for 3D-printing orthosis

For cases 1 and 2, we assessed the Jebsen–Taylor hand function test (JHFT), visual analog scale (VAS), and grasp and pinch power by a hand dynamometer for hand function before and after orthosis application. The JTHFT is a standardized measure for assessment of hand function that is designed to assess broad hand functions that are commonly used in activities of daily living.^[7]^ This timed test consists of seven functional tasks that require finger and hand movement. The test consists of the seven tasks of writing, turning over cards, picking up small objects, simulated feeding, stacking checkers, picking up large light cans, and picking up large heavy cans. The time to complete each of the 7 timed subtests was summed to produce a total JTHFT score. For case 3, we used a 6-minute walking test (6MWT) and modified Emory Functional Ambulation Profile (mEFAP) for ambulatory function. The 6MWT was done on a standardized course. Subjects walked for 6 minutes along a 30-m course with turns at each end; the course was marked every 3 m to allow accurate measurement of distance walked.^[8]^

The mEFAP consisted of 5 subtasks: a 5-m walk on a hard floor; a 5-m walk on carpet; a timed up and go; the navigation of a standardized obstacle course; and the ascent and descent of 4 stairs. The total mEFAP was calculated using the sum of the 5 subtasks.^[9,10]^ Stability score was measured during a standing posture using the Gaitview AFA-50 system (alFOOTs, Seoul, Republic of Korea) for balance function before and after AFO application. We used the Gaitview Pro 1.0 software to measure the stability score for balance function with eye opening.^[11]^ The higher score means better balance function.

In all cases, we also used the Quebec User Evaluation of Satisfaction with Assistive Technology (QUEST) of Korean version 2.0 after orthotics application. The QUEST is a self-report or interview-based scale, designed to evaluate a person's satisfaction with a wide range of assistive technology. The current version (ver. 2.0) covers satisfaction with both the device and the service from the vendor/manufacturer. Response categories range from 1 (not satisfied at all) to 5 (very satisfied). Administration time is approximately 10 to 15 minutes. The QUEST yields 3 scores: device, services, and a total QUEST, calculated by summing and then averaging valid responses to assigned items. The Korean version of QUEST 2.0 is reliable and valid for clinical use.^[12]^ All patients have provided informed consent for publication of the cases. Retrospective case reports were performed, which were approved by the Ethics Committee of Catholic Kwandong University International St Mary's Hospital.

## 3 Case presentation

### 3.1 Case 1: wrist orthosis for carpal tunnel syndrome

**Figure 1:**
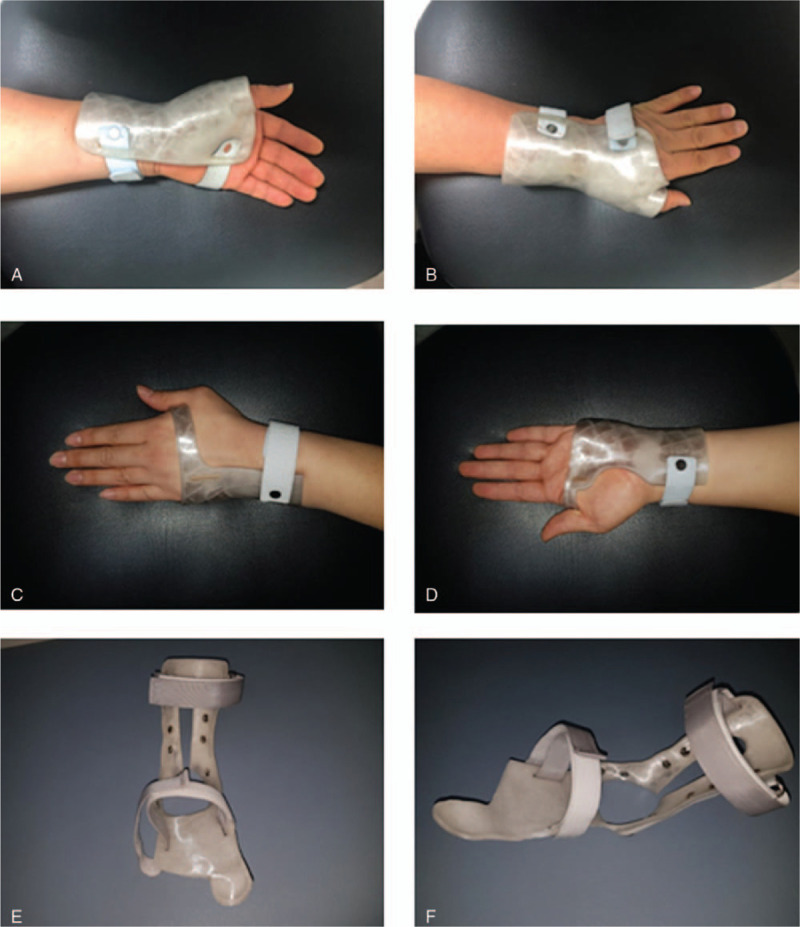
Wrist orthosis for carpal tunnel syndrome: (A) palmar view, (B) dorsal view. Ulnar wrist orthosis: (C) palmar view, (D) dorsal view. Ankle-foot orthosis: (E) anterior view, (F) lateral oblique view.

**Figure 2:**
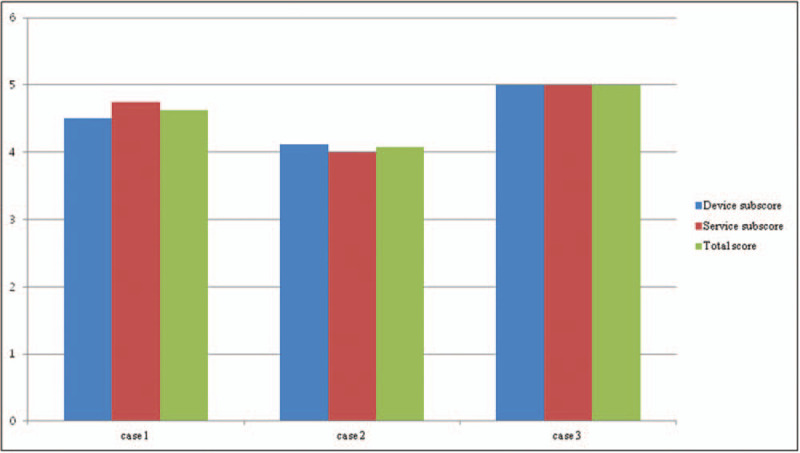
The score of Quebec User Evaluation of Satisfaction with Assistive Technology.

A 55-year-old male patient complained that the1st to 3rd fingers of his left hand (nondominant side) were tingling. Electrodiagnostic findings showed left median neuropathy at wrist level, a moderate carpal tunnel syndrome (CTS) according to the American Association of Electrodiagnostic Medicine classification.^[13]^ His left-hand grasp power was 16 kg, lateral pinch power was 5 kg, palmar pinch power was 4.5 kg, and tip pinch power was 5 kg by hand dynamometer. After 2 weeks of the use of wrist carpal tunnel orthotics (Fig. 1A, B), the grasp power was 22 kg, lateral pinch was 7 kg, palmar pinch was 4 kg, and tip pinch power was 6 kg. VAS score was decreased from 7 to 3. The mean score of JHFT was improved from 12.85 ± 1.77 to 14.12 ± 0.89 (Table 1). The Total QUEST score was 4.62 (Fig. 2).

**Table 1:**
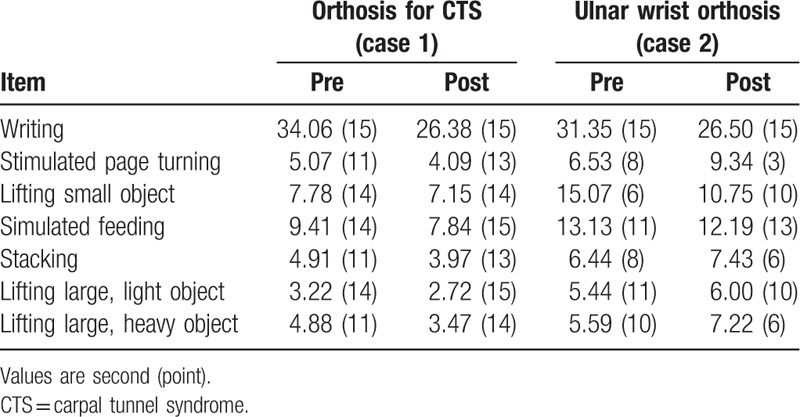
Jebsen–Taylor hand function test.

### 3.2 Case 2: ulnar wrist orthosis for ulnar neuropathy

Patient was a right-handed 59-year-old male having suffered from left wrist pain and 4th to 5th finger abnormal sensation. Before orthosis application, he underwent ulnar nerve neurolysis and an abscess was removed at the left wrist lateral aspect. After surgery, he was diagnosed with ulnar neuropathy around the wrist joint by electrodiagnosis. At that time, his left-hand grasp power was 12 kg, lateral pinch power was 4 kg, palmar pinch power was 4 kg, and tip pinch power was 3 kg by hand dynamometer. After 8 weeks of the use of ulnar wrist orthotics (Fig. 1C, D), the grasp power was 18 kg, lateral pinch was 3 kg, palmar pinch was 3 kg, and tip pinch power was 1 kg. The VAS score was improved from 6 to 4. The mean score of JHFT was decreased from 9.85 ± 2.91 to 9.00 ± 4.24 (Table 1). The total QUEST score was 4.08 (Fig. 2).

### 3.3 Case 3: ankle-foot orthosis for lumbar radiculopathy

A 72-year-old female patient complained of right foot drop. She underwent posterior lumbar interbody fusion and abscess removal surgery, 6 months before orthosis application. Her right-ankle dorsiflexor muscle strength had grade 2 on the Medical Research Council scale. She demonstrated hypoactive, patellar tendon reflexes on the right side. Imaging examination via magnetic resonance imaging on her lumbar spine showed fluid collection at the dorsal epidural space (L3 to L4 body level) and severe central canal compromise. Electrodiagnostic findings revealed right lower lumbar radiculopathy and spinal stenosis. She could walk and received gait training with 3D-printing manufactured AFO (Fig. 1E, F) for 4 weeks. The distance of the 6MWT was increased from 62.5 to 100 m. Total mEFAP score was improved from 182.97 to 104.07 seconds (Table 2). The stability score with eyes open was improved from 77 to 82, and the stability score with eyes closed was improved from 43 to 63. The total QUEST score was a perfect score, 5.00 (Fig. 2).

**Table 2:**
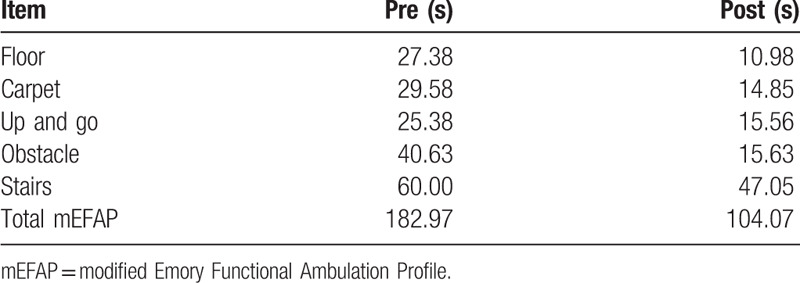
Modified Emory Functional Ambulation Profile (case 3).

## 4 Discussion and conclusion

Peripheral nerve injury or disease can cause symptoms of either partial or complete loss of sensory and motor function. Peripheral nerve entrapment describes the mechanical irritation by which a specific peripheral nerve becomes locally injured in a vulnerable anatomic site. Peripheral nerve entrapments produce focal disturbances of nerve function. There are several anatomical sites where peripheral nerves run in relatively confined spaces and are therefore at increased risk of compression. The median or ulnar nerve is usually involved around the wrist joint, called CTS, associated with median nerve or Guyon canal syndrome associated with ulnar nerve. The prescription, fabrication, and fitting of orthosis require a sufficient understanding of the basic mechanical principles of splinting; knowledge of the mechanical properties of splinting materials, of deep and surface anatomy, and of the effect of compressive tensile and shear forces on the integument, and a thorough understanding of the pathophysiology, diagnosis, and treatment of peripheral nerve injury.

The CTS is the most prevalent entrapment neuropathy of the upper extremity and is described by the presence of a variety of neurologic signs and symptoms resulting from the localized compression of the median nerve in the carpal tunnel.^[14]^ A diagnosis is based on the patient's history and a clinical examination. Electrodiagnostic examination can usually confirm the clinical diagnosis.^[15]^ Wrist orthosis is widely used in treatment of CTS besides other conventional treatment options, including surgery, nonsteroidal anti-inflammatory drugs, and steroid injections.^[16]^ The rationale for using a wrist orthosis was originally based on observations that CTS symptoms improve with rest and worsen with activity. Subsequent research has suggested that the therapeutic effect of using a wrist orthosis arises from minimizing carpal tunnel pressure,^[16]^ which is strongly concerned in the pathophysiology of CTS, and pressure in the tunnel increases with wrist positions away from neutral.^[16]^ A Cochrane database systematic review about the use of orthotic devices for CTS was previously published.^[17]^ In the literature, conservative treatment is preferred in mild to moderate cases of CTS, whereas surgical treatment is mainly applied in severe cases. However, surgical treatment is also indicated when conservative management fails for patients with CTS.^[18,19]^ In case 1, this patient was diagnosed as moderate CTS by electrodiagnositic examination. Most of his hand function improved after application of 3D-manufactured CTS orthosis.

Ulnar neuropathy at the wrist and hand can range from pure sensory to pure motor deficits.^[20,21]^ Guyon canal syndrome is a relatively rare peripheral neuropathy which involves injury to the distal portion of the ulnar nerve as it travels through a narrow anatomic corridor at the wrist. Guyon canal is a unique location where the ulnar nerve is vulnerable to compressive injury, although the more common location of the ulnar nerve injury is at the elbow, which is known as cubital tunnel syndrome. The most common type of ulnar neuropathies at the wrist is compression of the deep palmar branch.^[21,22]^ Ulnar neuropathies of the wrist and hand are divided into 3 types. Type I is a lesion of the ulnar nerve just proximal to or within the Guyon canal involving deep and superficial branches; this causes mixed motor and sensory deficits and subsequent weakness of all the ulnar hand muscles. Type II is a lesion involving the deep branch, which causes a pure motor deficit with a varied pattern of weakness based on the compression site. Type III is a lesion limited to the superficial branch, causing purely sensory deficits to the palmar aspect of the medial half of the 4th digit and the 5th digit^[23]^ (https://www.ncbi.nlm.nih.gov/books/NBK534226/). The most commonly reported cases of Guyon canal syndrome result from a ganglion cyst and repetitive trauma.^[21]^ The decision to choose conservative vs surgical management depends on the duration and severity of symptoms, as well as the exact etiology found to be the cause of the symptoms. Wrist splinting must keep the wrist in neutral a position but allow fingers to move around freely. A splint is to be worn at least at night for a recommended duration of 1 to 12 weeks.^[24]^ However, there are opposite opinions. According to the European Hand guide study published in the British Medical Journal, the surgical decompression was favored for moderate to severe symptoms lasting 3 months or longer. Postsurgical splinting was not a necessity but could be used in those patients who have a habit of loading the wrist joint.^[22,25]^ In our case, we designed with reference to the ulnar gutter splint, which is usually used after wrist surgical operation. However, most hand functions were not improved except for pain severity, probably because of an insufficient wearing period. And it is possible that the patient was not a suitable indication for ulnar gutter splint.

Complex injuries to the lower extremity and spine may involve the lumbosacral root or plexus and sciatic or peroneal nerve damage resulting in partial paralysis, manifesting in loss of ankle dorsiflexion and eversion known as a “drop foot” pathology.^[26]^ The resulting motor and proprioceptive deficits lead to walking and running difficulties in spite of compensatory mechanisms, such as increased knee and hip flexion, because the toes drag and cannot clear the ground during the swing phase of the gait cycle.^[27]^ AFOs are the most common treatment for drop foot of the lower extremity. An AFO is designed so that the limp foot is supported by being coupled to the lower leg.^[28]^ An AFO is also traditionally prescribed for hemiplegic patients after stroke or head injury, paraplegic patients with a spinal cord injury, and flaccid paretic patients with foot drop.^[29]^ The traditional thermoplastic flexible AFO is composed of a molded sheet of plastic that wraps around the posterior of the leg and under the foot with fabric straps across the ankle to secure the heel in place. The brace is most often made from polypropylene or polyethylene plastic. An articulated AFO retains the solid foot piece and leg piece of the standard AFO, but flexes at small ankle joints built into the brace.^[30]^ A foot drop associated with peripheral nervous injury is different from the central nervous system injury. Because there is no spasticity in a peripheral nervous system injury, an AFO for peripheral nerve injury is simpler than an AFO for central nervous system injury. The ankle-joint component of an AFO for peripheral nerve injury is enough for the leaf-spring action of the brace itself. In our case, we proved that the 3D-manufactured AFO could improve walking speed, bring gait characteristics closer to functional ambulation, and provide enough stability, dynamic support, or freedom of movement to allow nearly normal function.

The use of wrist orthosis allows unhindered continuation of the existing work and maintains the durability of work. Mass-produced, ready-made orthoses are of lower quality are bulky, and are uncomfortable to wear compared to custom-made wrist orthoses. However, custom-made wrist orthoses are more expensive than ready-made ones and take a long time to produce. The 3D printer technology can overcome these problems by producing personalized medical products with low cost and reduced time.^[31]^ 3D-printing data files are also advantageous for the production of individualized products at no extra cost, even when different designs are used in consecutive production runs. In addition, because the 3D printer is controlled by a computer, it can produce objects in various forms and is easier to use than are other manufacturing techniques. 3D printing is a manufacturing method whereby materials are joined, layer by layer, to fabricate an object from a digital source. It has the potential to eliminate several steps associated with traditional methods. 3D printing enables design freedom by facilitating deviation from traditional design paradigms and hence allows the development of patient-specific orthosis.^[4,32]^ In our case, the 3D-printed wrist orthosis was personalized by using CT-scanned 3D images and was printed out using thermoplastic polyurethane filament by a 3D printer. An AFO manufactured by 3D printing had a unique design considering a progress direction of foot center of pressure during gait cycle. This orthosis could be optimized to individual biomechanical requirements to provide improved function, better fit, and better aesthetics. The QUEST of Korean version 2.0 for satisfaction was evaluated after orthotics application. In QUEST items, all 3D-printed devices showed high scores (more than 4 out of 5 points). On the whole, the patients with peripheral nervous system injury were satisfied with the 3D-manufactured orthosis. Conclusively, we designed and manufactured a patient-specific assistive device through 3D-printing techniques optimized for patient function after estimating the disability status of a patient with peripheral nerve injury. We hope to provide effective personalized orthosis to disabled patients using 3D-printing techniques. In the future, a comparative study with conventionally made orthosis and a larger sample would be necessary.

## Acknowledgments

The authors thank all the patients for their contribution to the study. The authors are grateful to the colleagues in the 3D-printing center of the Korean National University of Transportation for providing them with case record support and also thank HARRISCO for English editing services.

## Author contributions

**Conceptualization:** Dong-Sik Chae, Jae-Hyung Kim.

**Data curation:** Da-Ham Kim, Kyung-Yil Kang.

**Resources:** Sung-Jun Park.

**Writing – original draft:** Dong-Sik Chae, Jae-Hyung Kim.

**Writing – review & editing:** Doo-Young Kim, Si-Woon Park.

Jae-Hyung Kim orcid: 0000-0001-8713-0489.
